# ACES: Analysis of Conservation with an Extensive list of Species

**DOI:** 10.1093/bioinformatics/btab684

**Published:** 2021-10-02

**Authors:** Evin M Padhi, Jeffrey K Ng, Elvisa Mehinovic, Eleanor I Sams, Tychele N Turner

**Affiliations:** Department of Genetics, Washington University School of Medicine, St. Louis, MO 63110, USA; Department of Genetics, Washington University School of Medicine, St. Louis, MO 63110, USA; Department of Genetics, Washington University School of Medicine, St. Louis, MO 63110, USA; Department of Genetics, Washington University School of Medicine, St. Louis, MO 63110, USA; Department of Genetics, Washington University School of Medicine, St. Louis, MO 63110, USA

## Abstract

**Motivation:**

An abundance of new reference genomes is becoming available through large-scale sequencing efforts. While the reference FASTA for each genome is available, there is currently no automated mechanism to query a specific sequence across all new reference genomes.

**Results:**

We developed ACES (Analysis of Conservation with an Extensive list of Species) as a computational workflow to query specific sequences of interest (e.g. enhancers, promoters, exons) against reference genomes with an available reference FASTA. This automated workflow generates BLAST hits against each of the reference genomes, a multiple sequence alignment file, a graphical fragment assembly file and a phylogenetic tree file. These data files can then be used by the researcher in several ways to provide key insights into conservation of the query sequence.

**Availability and implementation:**

ACES is available at https://github.com/TNTurnerLab/ACES

**Supplementary information:**

Supplementary data are available at *Bioinformatics* online.

## 1 Introduction

Recently, long-read sequencing and other advanced genomic technologies have enabled cost-effective reference genomes in several species (Rhie *et al.*, 2021). Exemplar projects, such as the Vertebrate Genomes Project (Rhie *et al.*, 2021), are generating high-quality reference genomes for all vertebrate species. This powerful dataset will provide key insights in numerous areas. An area of interest in the study of human disease is to assess the conservation of specific elements in the genome (e.g. enhancers, promoters, exons), especially those that occur outside of the protein coding regions of the genome.

In performing characterization of various genomic elements, we sought to use these new reference genome resources to gain deeper insights about our elements of interest. However, there was no tool to quickly generate data for a comparative genomic analysis using these new genomic datasets, resulting in a bottleneck in our work.

To alleviate this problem, we developed an automated workflow utilizing BLAST to query sequences across all reference genomes and ultimately generate a multisequence alignment file, graphical alignment file and phylogenetic tree files. Our pipeline additionally provides a ready to use database of genomes for deep comparative analyses of reference genomes from the Vertebrate Genomes Project and Ensembl.

One interesting example, which also served as a benchmark for our workflow, that we focus on in the present study is the ZRS enhancer that targets the *SHH* gene and is involved in preaxial polydactyly (Lettice *et al.*, 2003). This element is highly conserved in vertebrates but has lost its function in snakes due to a 17 nucleotide deletion in a specific transcription factor binding site that has been functionally validated to modulate limb development (Kvon *et al.*, 2016).

## 2 Application

Analysis of Conservation with an Extensive list of Species (ACES) is a computational workflow that performs evolutionary analysis of a given nonrepetitive query sequence under 3 kb pairs, in FASTA format, by comparing it to genome data from any reference genomes of interest to the user provided sequence. A schematic of the workflow is shown in Supplementary Figure S1.

ACES starts by taking a sequence of interest and performs a BLASTn (version 2.5.0+) (Altschul *et al.*, 1990) search for distant homologs (dc-megablast) on each genome which has been converted to a blast database to reduce runtime and storage. Using a search strategy designed to identify distantly related orthologs, it returns the best sequence meeting user defined e-value, length and percent identity thresholds while simultaneously recording the hits in a log file. ACES by default takes the best sequence identified but has parameters to allow for handling of multiple orthologs per species. Next, ACES performs a multiple sequence alignment using MUSCLE (v3.8.1551) (Edgar, 2004) to generate both a multiple sequence alignment file and PHYLIP output files. Then, the multiple sequence alignment file is run through a python program (our modified version of https://github.com/fawaz-dabbaghieh/msa_to_gfa version 1.0.0) to convert the multiple sequence alignment file to a graphical fragment assembly (GFA) file. A GFA file is a tab-delimited text file that describes sequences and their overlaps. With the help of an outside GFA viewer [e.g. BANDAGE (Wick *et al.*, 2015)], the user can import the generated GFA file to view the sequence graph and visualize conservation. ACES next utilize RAxML (version 8.2.12) (Stamatakis, 2014) to generate a phylogenetic tree file and estimate the rates of evolution at the locus of interest. By default, ACES uses the GTRGAMMA model of evolution, as it is one of the most general models for nucleotide substitution and allows for varying rates of substitutions, and performs bootstrap for maximum likelihood (ML) trees. Once finished, the tree file with the best likelihood is generated along with four supporting RAxML files that can be used in downstream evolutionary analysis. We note that while ACES generates these results, the researcher should carefully consider each of these outputs in the context of their study, in particular, the bootstrap results [see discussion on this in Russo and Selvatti (2018)]. Finally, the ACES wiki provides instructions on how to use the PHAST package (Siepel *et al.*, 2005) to produce a per-base score that represents the probability that a given base is belongs to a conserved element and also identify discrete conserved elements.

## 3 Results

We tested ACES on a sequence located within the ZRS enhancer region (https://www.ncbi.nlm.nih.gov/gene?Db=gene&Cmd=DetailsSearch&Term=105804841). Specifically, we tested a 766 nucleotide subset of this sequence that had previously been examined and shown to contain a 17 nucleotide deletion in snakes (Kvon *et al.*, 2016). When this deletion is introduced in mice it promotes a reduced limb growth phenotype. We sought to determine whether our fully automated approach could recapitulate the results in the Kvon paper. We aggregated the same 18 reference genomes, as found in the Kvon paper, and utilized our default settings of a BLAST e-value threshold of 0.00001 and a query length fraction of at least 0.3. After all the files were generated by ACES, including a multiple sequence alignment file, best ML tree file and a GFA file, we proceeded to characterize the output files using existing downstream programs.

**Fig. 1.**
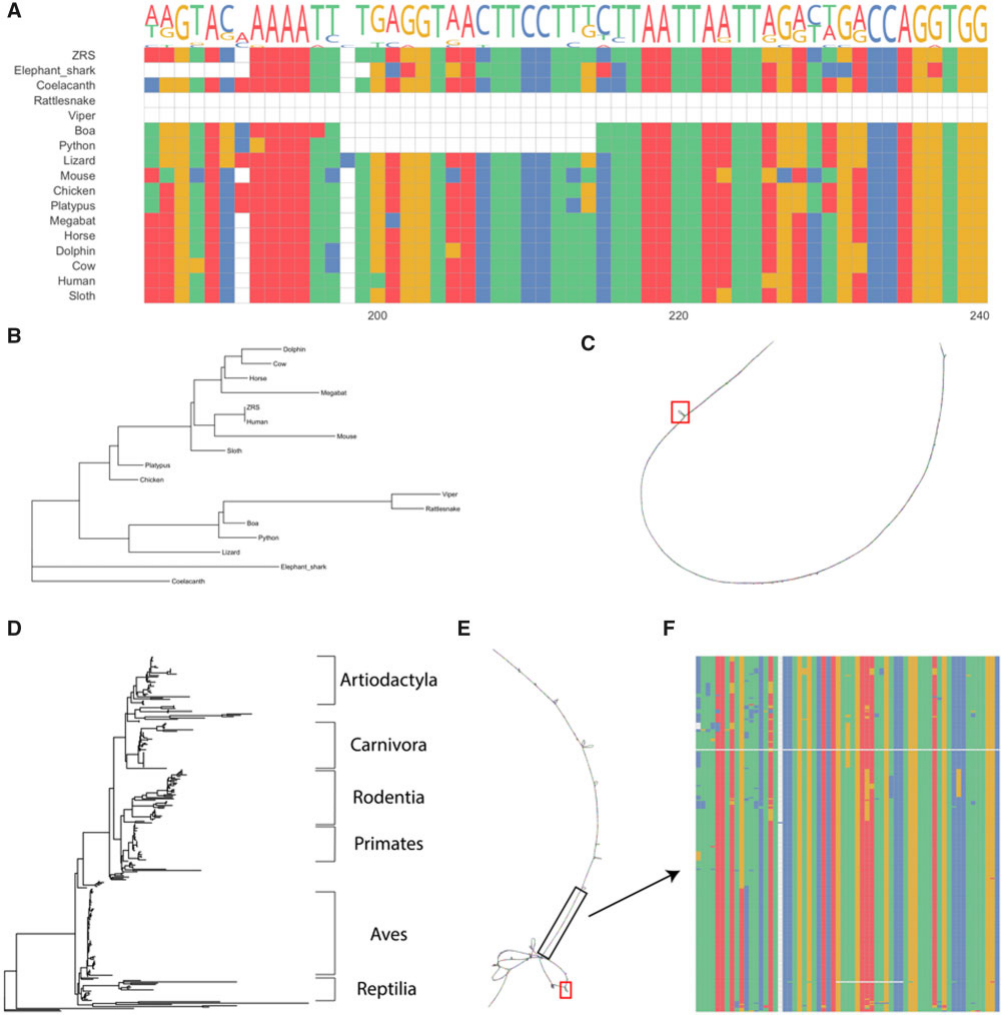
Analyses of the ZRS enhancer with ACES. (**A**) Alignment of the ZRS sequence used as a query in ACES and the 16 species used in Kvon *et al.* (2016) highlighting a previously found snake-specific deletion. (**B**) Phylogenetic tree generated using ACES with all species shown in A. (**C**) Bandage plot of the alignment in A, red box indicates the location of the snake-specific deletion. (**D**) Phylogenetic tree generated using ACES with VGP and ENSEMBL reference genomes for ZRS. (**E**) Bandage plot of the ACES ZRS VGP-ENSEMBL alignment, black box indicates a conserved element identified by PhastCons, red box indicates snake-specific deletion identified in A. (**F**) Multiple sequence alignment of the highlighted conserved element in E

First, we assessed the multiple sequence alignment in MEGA (Kumar *et al.*, 2016) and observed the expected 17 nucleotide deletion in snakes (Fig. 1A). We then examined the best tree and found that the snakes all cluster together (Fig. 1B) and also represented this in a BANDAGE plot using the GFA file to show that most of the element is conserved aside from a few small regions (Fig. 1C). This replicates the findings in Kvon *et al.* (2016), suggesting that the pipeline produced the expected outcome. Running locally on a computer with an Intel i7-8700 @ 3.20 GHz and 64 GB of RAM, this test case ran in around 1 min and 30 s. In the cloud, run time was about 20 min. This longer time, in the cloud, was due to the population of the data in the cloud workspace.

To further demonstrate the utility of ACES, we performed a comprehensive analysis of the same sequence using all the currently available Vertebrate Genomes Project and Ensembl release 103 genomes (Howe *et al.*, 2021) (*n* = 393). This ran in 2 h and 20 min in the cloud and we again examined the output data to see the full phylogenetic tree (visualized using icytree.org in Fig. 1D). The BANDAGE plot of this analysis revealed a more complex diagram of the conservation of this element (Fig. 1E). On average, our branches in the phylogenetic tree file had a bootstrap value of 62.5 with running 100 bootstraps. Using PHAST, we also identified a highly conserved sequence in this element (Fig. 1E).

## 4 Discussion

Reference genome data are being generated rapidly and our field is on the path to new biological discoveries with this diverse data. We identified a current gap caused by the new data, which is the ability to utilize all of this data to quickly look at specific regions of interest. To address this gap, we developed ACES as a fast workflow to query sequences of interest and derive a multiple sequence alignment file, best ML tree file and GFA file for each sequence of interest. ACES is flexible and allows for testing of any number of reference genomes, which will be a boon as many new genomes continue to become available in the coming years. This workflow will be of interest to individuals assessing small sequences under 3 kb pairs (e.g. enhancers, promoters, exons) across many genomes and useful in identifying new regions for downstream analyses and functional investigation. To facilitate usage of ACES we have set up a code base (https://github.com/TNTurnerLab/ACES), with both local and cloud-based instructions, and have provided small test datasets for users to access.

## Data availability

Reference genomes are publicly available at the Ensembl website at http://ftp.ensembl.org/pub/ and the specific versions used in this paper are also available on the Turner Lab Public Globus Endpoint at https://app.globus.org/file-manager?origin_id=97668938-bcc8-11eb-9d92-5f1f6f07872f&origin_path=%2F.

## Supplementary Material

btab684_Supplementary_Data
