# Supplementary figures and images for: ACES: Analysis of Conservation with an Extensive list of Species
Source: Bioinformatics. 2021 Oct 2;37(21):3920–2. doi: 10.1093/bioinformatics/btab684 (PMC8570785; doi:10.1093/bioinformatics/btab684)

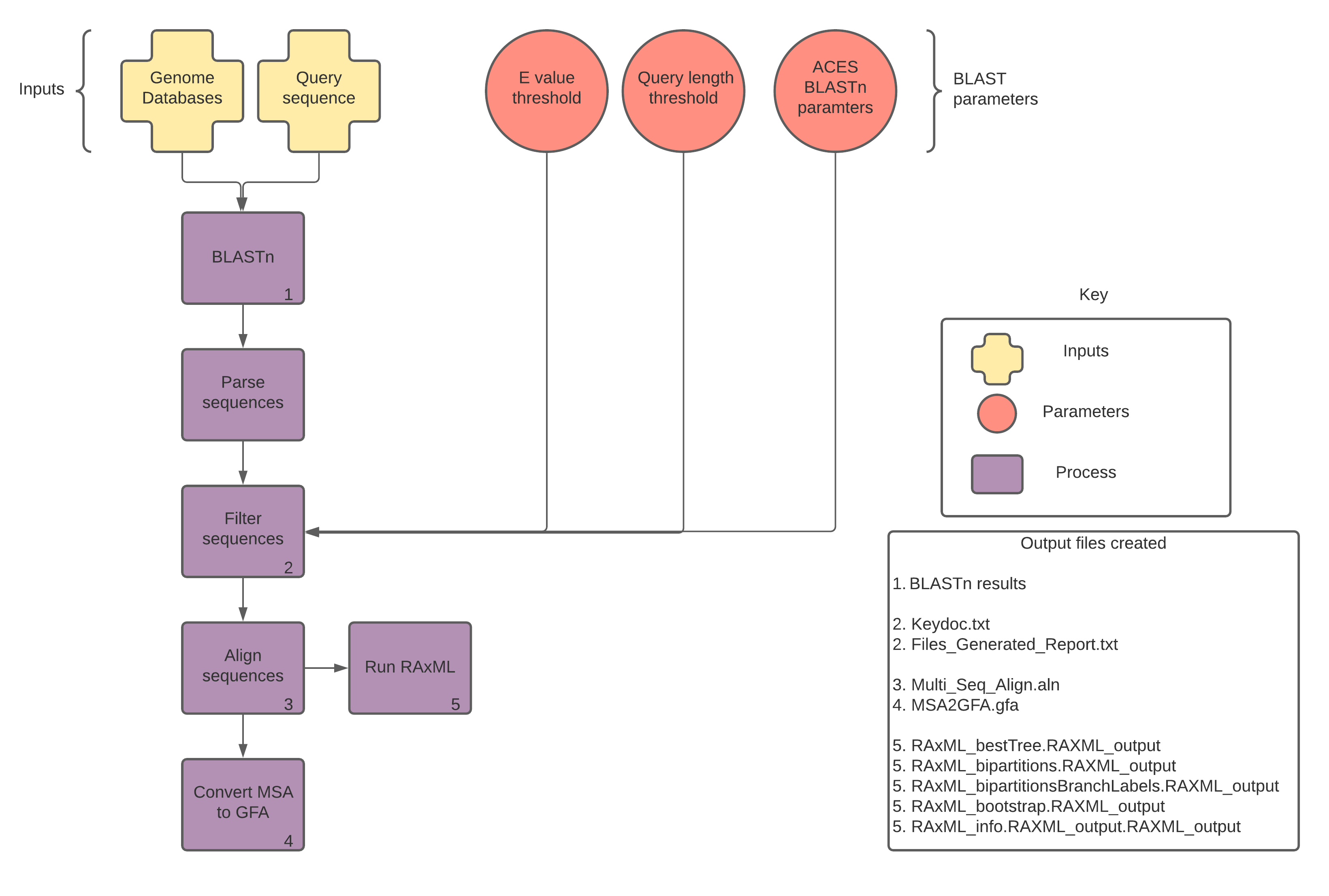

Supplement: btab684_Supplementary_Data [file btab684_supplementary_data.zip › btab684_Supplementary_Data.png]
